# The Impact of the COVID-19 Crisis on the Practices and Mental Health of Psychologists in Belgium: Between Exhaustion and Resilience

**DOI:** 10.3390/ijerph192114410

**Published:** 2022-11-03

**Authors:** Fabienne Glowacz, Emilie Schmits, Annabelle Kinard

**Affiliations:** Department of Psychology-Adaptation Resilience and Change Research Unit (ARCh), University of Liege–Belgium, Place des Orateurs, 1-B33, 4000 Liège, Belgium

**Keywords:** COVID-19 pandemic, psychologists, mental health, burnout, innovative practices

## Abstract

While the COVID-19 pandemic has created psychological distress in the general population and increased the need for psychological care, little research has been done on how mental health practitioners (MHP) have been affected by the pandemic, and these health professionals have received little attention from public authorities. In this article, we focus on psychologists and the impact that the pandemic has had on their mental health and practices by exploring the adaptive and innovative responses generated. This study is based on an online survey (including multiple choice questions, several validated scales, and eight free text items) completed by 187 psychologists (86% female) one year after the beginning of the COVID-19 pandemic in Belgium (February–April 2021). Most participants considered that the crisis had an impact on their well-being and mental health. However, the prevalence of symptoms of depression and anxiety was relatively low (17%; 12%). On the other hand, the majority of psychologists (72%) suffered from a medium level of burnout (BO), 7% suffered from a high level of BO, and only 21% had low levels of BO. Psychologists working in face-to-face settings had the highest scores on the “exhaustion” subscale of the BO, and those working primarily with patients in precarious situations had significantly higher scores of BO and exhaustion. Qualitative analysis of free text items showed that MHP were resilience and developed new frameworks and modes for proactive interventions in order to reach their patients, meet the psychological and social population’s needs, and maintain their relationships with the network. In a crisis or pandemic context, public policies should take into account the psychological and social needs of the most socially precarious populations in reinforcing and supporting mental health professionals working in this sector.

## 1. Introduction

Coronavirus disease (COVID-19) is an infectious disease caused by the SARS-CoV-2 virus. It was identified as the causative agent for a series of atypical respiratory diseases in the Hubei Province of Wuhan, China, in December of 2019 [1]. The infectious disease, termed COVID-19, was officially declared a pandemic by the World Health Organization on 11 March 2020 [2]. Governments across the world imposed restrictive measures, such as personal measures (e.g., hand hygiene; use of masks); physical and social distancing measures (e.g., physical distancing; homework; lockdowns); and movement measures with limiting movement of persons locally or nationally (WHO, 2020). Belgium, like many European countries, was heavily affected by the COVID-19 pandemic. It was among the first European countries to implement nationwide containment measures at several moments throughout the pandemic [3]. The federal government ordered a first lockdown from March to May 2020, with a complete closure of schools, health services (except hospitals), restaurants and shops. From May 2020, deconfinement strategies were put in place, including the gradual opening of services. Care institutions resumed their activities according to the guidelines of their institution’s management (face-to-face, virtual, teleworking, etc.). A second partial lockdown was declared by the federal government from October 2020 to April 2021, which targeted restaurants, bars, and schools in particular. The care services continued their activities according to an adaptation of the health measures recommended by the government and implemented by their respective management. 

The COVID-19 pandemic and the measures implemented to deal with the virus disrupted the social and professional lives of people the world over and had major implications for mental health [4]. However, during the first year of the pandemic, mental health and psychosocial care received little attention from politicians and the wider scientific community. This can be understood by the predominance of the “hospital-centric” approach taken by decision-makers during this crisis, where one of the reference criteria was the capacity of hospitals to respond to the spread of COVID-19. One year after the COVID-19 pandemic, numerous international research studies [5,6,7,8,9] have identified the effects of the crisis and the health measures that had been implemented as a result of the mental health of the general population for Belgium [10] and of health professionals [3,11,12,13]. However, one area remains poorly documented, that of mental health practitioners (MHP) [11].

### Impact of the Crisis on Mental Health Practitioners (MHP)

The psychological distress of the population in the early months of the pandemic, which was observed worldwide, led to an increased demand for mental health care services and emphasized the critical roles played by MHP, such as psychologists, social workers, psychosocial counselors, and psychiatrists during this health crisis [5]. However, the COVID-19 pandemic also had consequences for the medico-psychosocial care system, both for caregivers and intervention modalities. Indeed, MHP were, and still are, faced with the complex challenges of managing psychological distress, the potential increase in professional mandates, and organizational adaptations made in response to the crisis, while also having to deal with their own fears and stressors related to COVID-19 [14]. It must be noted that the mental health of these professionals has received little public or scientific attention [15,16,17,18,19,20], which may seem paradoxical in light of the high levels of psychological distress among the population since the start of the COVID-19 pandemic [21,22,23]. 

In the early months of the COVID-19 pandemic, scientific papers considered the possibility that frontline health care workers might be affected by post-traumatic stress, anxiety, depression, and burnout [24]. Primary care providers in hospitals have indeed been exposed to many stressors, such as repeated exposure to severely infected and dying people, intense and stressful work environments, and busy schedules that can lead to burnout. Many systematic reviews and meta-analyses confirm a high prevalence of depressive symptoms, anxiety, insomnia, psychological trauma, fatigue, distress, and burnout among primary care providers [11,12,13,25]. A recent meta-analysis conducted by Saragih et al. [26], including 38 studies, found mental health prevalence for post-traumatic stress disorder, anxiety, depression, and distress to be 49% (95% confidence interval [CI]: 22–75%), 40% (95% CI: 29–52%), 37% (95% CI: 29–45%), and 37% (95% CI: 25–50%), respectively. According to these studies, women, young professionals, and non-physicians, in particular, face a higher risk of mental health problems and burnout. Other risk factors highlighted by these same studies include primary care status, fear or risk of infection, current or past mental health problems, and low social support [12,13,16,25,27]. 

During disasters, pandemics, and other public emergencies, social workers and psychologists provide essential services to the public. In the context of the COVID-19 pandemic, they have an ethical mandate to serve populations that are most vulnerable to the negative consequences associated with COVID-19 [28,29]. Indeed, the current pandemic and the measures put in place to counter it have challenged mental health care staff to provide counseling and support to children, families, and more vulnerable populations under conditions that limit their usual flexibility and practices [15,28]. For many practitioners, working in the context of the pandemic has caused considerable stress [29]. To date, few studies have specifically considered how the pandemic has affected the mental health of psychologists. One study focused on psychotherapists, finding that younger, less experienced therapists were at greater risk for vicarious trauma [30]. Another study aimed at psychotherapists, which was conducted in the first weeks of the COVID-19 enforced lockdown in Austria, found higher levels of stress than in a representative sample, and even more so when psychotherapy was the research subject’s only income [31]. A more recent work carried out in Quebec that compared the psychological distress of MHP and workers from the general population found a lower prevalence of clinically significant depression (19% versus 27%) or anxiety (16% versus 29%) among MHP, but with an increase in the levels of depressive and anxiety symptoms in regions with high incidences of COVID-19 cases [14]. These initial publications concluded that there is a need for further research to better identify what supports resilience or, conversely, how it affects the mental health of psychologists. Specific studies related to psychologists have mainly focused on the transition to virtual interventions and practitioners’ adoption of telepsychology during the crisis [32,33,34].

Social distancing guidelines that have promoted telecommuting have fundamentally changed the way psychologists conduct their counseling sessions, which may also have been an additional stressor [19]. Pierce and colleagues’ [32] survey of 2619 psychologists indicated that 96% used telepsychology (i.e., online psychology (telephone psychology, internet psychology)) at least to some extent after the onset of the crisis, and 78% reported using it for 90–100% of their practice. Research by Sammons (2021) confirmed the continuation of these practices 6 months into the pandemic, and even noted an increase in services provided through telepsychology [33]. 

According to this study, most psychologists considered that their patients had appropriate access to the internet and telepsychology service platforms, but one-fifth of patients had difficulty accessing these services. While before the crisis, telepsychology practices were occasional and often confusing [35], they became common practices during the crisis, with many psychologists stating that they would continue to use them for the delivery of some of their psychological services after the pandemic had ended [34].

However, the impact of the crisis on psychologists’ well-being and practices has not yet been fully explored, nor has the deployment of new practices (other than tele-psychology). Moreover, no study, to our knowledge, has considered the profiles of a patient’s population to evaluate the mental health of MHP. Given this stark lacuna in the literature, we conducted an online survey after one year of crisis with the objective of collecting scientific data and evaluating the mental health (depression, anxiety, and Burnout) and the associated practices of psychologists in exploring the adaptive responses generated in the face of the crisis. We hypothesized that, after one year of crisis, the prevalence of depression, anxiety, and BO would be high. More specifically, the modalities of work during the crisis (face-to-face or remote) and the social profile of the patients could differentiate the mental health and BO of psychologists. Finally, as any crisis causes significant difficulties for health professionals but can also stimulate innovative practices, we will explore in this study the difficulties/problematic situations and the motivating/positive situations in the professional environment and practices developed during the COVID-19 crisis.

## 2. Materials and Methods

### 2.1. Participants

This study focused on a sample of 187 psychologists who were part of a larger study of psycho-social workers from different professions (N = 784). Samples were only composed of participants who completed more than 80% of the questionnaire and were retained. Belgian French-speaking psychologists and practicing in the Wallonia or Brussels region could be included in this study and responded to all questions (after signing a consent form) of an online self-report questionnaire (approved by the ethics committee of the University of Liege). The survey was distributed widely by email and online through social networks such as Facebook and LinkedIn (between February and April 2021) in the private and public sectors with different missions to the population: medical care, mental health care, justice, education, community, and youth protection. No obligation to participate or remuneration was provided. 

### 2.2. Measures

The online questionnaire is composed of forty-six questions in French with a duration of about 20 min, including multiple choice questions, several validated scales, and eight free text items. This survey was available on a personal online survey system designed and managed by the IT department of the Faculty of Psychology at the University of Liege (UDI-FPLSE). 

#### 2.2.1. Sociodemographic and Professional Data

The first questions focused on the personal life context (marital status, children, housing) and vaccination intention of psychologists. In addition to basic socio-professional data (professional status, work time, experience), the type of professional support (e.g., within the work structure itself, in the patients’ living environment), the target population (children, adolescents, or adults), the socioeconomic status of the patients (diverse, moderately precarious, very precarious), and the work modes (remote, face-to-face, and hybrid interventions) were assessed. 

Work arrangements during the crisis were identified at three time periods: the first lockdown from March to May 2020, the in-between lockdown period from June to September 2020, and the second partial lockdown from October 2020 until April 2021. 

#### 2.2.2. Variables Related to Mental Health 

Six questions addressed the participants’ subjective assessment of their well-being, and two validated scales were proposed. Burnout over the past seven days was assessed using the Oldenburg Burnout Inventory (OLBI) consisting of 16 items with a Likert scale ranging from 1 “strongly disagree” to 4 “strongly agree”. This scale assesses two dimensions of BO with 8 items for exhaustion and 8 items for disengagement [36,37]. The maximum score for each of the exhaustion and disengagement subscales is 32 points. The total score for BO is 64 points. Depression and anxiety over the last month were assessed by the HAD scale (Hospital Anxiety and Depression scale) [38], which has been used in other studies on the impact of the COVID-19 pandemic on mental health in the general population in Belgium and is a measure that proposes seven items relating to depression and seven items to anxiety [10,39]. All cutoff scores linked with these two scales are displayed in Table 1. 

In line with the work of Hansez [40], participants had to respond to three open-ended questions, namely free text response items, to describe 1 to 3 problematic/stressful situations and 1 to 3 motivating situations experienced at work during the COVID-19 period. Finally, participants answered an additional item about the perceived impact of the crisis on their well-being (“Have you noticed any changes in your well-being and/or difficulties since the beginning of the crisis?”). 

### 2.3. Data Analysis: Quantitative and Qualitative Data

SPSS 27 software (SPSS Inc., Chicago, IL, USA) was used to perform descriptive and inferential statistics (one-way analysis of variance). Because group sizes were not equal and the homogeneity of variance was not assumed (*p* < 0.001), the nonparametric equivalent of a one-way analysis of variance between groups was performed to make comparisons, namely Kruskal-Wallis one-way analysis of variance and Bonferroni corrections for post-hoc. Statistical significance was set at *p* < 0.05. 

For qualitative data, a thematic analysis was conducted to identify, analyze, and interpret patterns of meaning (‘themes’) [41,42]. A theme is a precise name in relation to the content of a corpus extract. The researcher has to assign themes to answer a research question by favoring low inference and close to the text. In this study, each participant could give up from one to three situations as answers per each of the three open questions about difficulties and motivating situations (‘Name 1 to 3 motivating and/or positive situations you have experienced in your work since the COVID-19 pandemic.’) and practices developed during the COVID-19 pandemic (‘Could you describe 1 to 3 practices that you have developed since the COVID-19 pandemic?’). Each participant could give up from one to three situations as answers to each of the three open questions. We have added two question headings to improve our understanding. We have coded all responses. We decided in this article to present only answers obtained for the first situation at each question because they take up almost all the emerging themes, and the responses to situations 2 and 3 only reinforce the validity of our thematisation. This analysis focused only on the first answer given by participants to each question. Two researchers read each answer and attributed the codes to an Excel document. After that, the analysis step consisted of creating themes based on codes with identical or similar meanings [43,44]. 

## 3. Results

### 3.1. Socio-Professional Characteristics

The sample is composed of 187 psychologists aged between 22 and 72 (M = 41.6; SD: 10.7), of which the majority are women (86%), as is the case for this population in Belgium [45]. Psychologists in this study have professional experience ranging from 1 to 46 years (M = 15.2; SD: 10.4) and are employed in services with different missions to the population: mental health centers, mobile teams, psychosocial services, hospitals, penal institutions, and youth protection services.

### 3.2. Working Environment and Intervention Methods during the Crisis

This sample reflects a diversity of professional contexts, with more than half of these professionals mainly practiced in their respective service’s offices before the COVID-19 pandemic. Information was retrospectively requested on the working modes of participants (on site, teleworking, both, off work) three times during the pandemic (the first lockdown between March and May 2020; between the two lockdowns, from June to September; and the second lockdown between October 2020 and April 2021) (see Table 2). During these two lockdown periods, teleconsultations have been widely used between health care professionals and patients with platforms such as Zoom, Skype, or Teams. Since October 2020, very few respondents (2%) maintained exclusively remote interventions (including teleconsultations and contacts by email or telephone with patients), while 65% conducted their interventions face-to-face (in direct contact and with barrier gestures), and 33% alternated between remote and face-to-face activities. 

Half of psychologists reported perceiving any change in the number of patients during the COVID-19 pandemic, while 13% noted a decrease and 37% an increase. 

More than half (63%) of the psychologists worked with adults, and the other half (37%) worked with children and teenagers. Half of the psychologists work with patients perceived as belonging to various socio-economic levels, and the other half with patients who are moderately precarious and very precarious socially and economically (see Table 3).

### 3.3. Depression, Anxiety and Burnout

The evaluation of rates of depression, anxiety, and burnout was based, first, on the subjects’ perceptions of the effects of the crisis on their mental health and then on the validated measures (HAD and OLBI). Almost all respondents perceived negative effects related to the pandemic on their mental health. These effects were perceived as slight for 65%, but 29% found them considerable; only 3 subjects reported no negative effects related to the crisis. In the second question concerning the perceived change in well-being, some nuances were perceptible: 38% of the psychologists did not perceive any change in their well-being and difficulties since the beginning crisis, 11% felt more motivated and in a better mood, and half (51%) declared themselves discouraged and demotivated. Although this self-assessment reflected the negative impact of the crisis on the well-being of more than half of the practitioners, one in 10 practitioners reported feeling more motivated. 

This tendency was even more evident when participants were asked about the influence of the crisis on their career; more than half of the psychologists (53%) said they were more committed to their work as psychologists (32% said they were not affected by the crisis), while 14% of the sample said they were reconsidering their career choice. 

Regarding anxiety and depression, the results showed that most of the respondents respondents did not report depressive and anxiety symptoms (61%; 64%), and the prevalence of symptoms of depression and anxiety was relatively low (17%; 12%). For burnout, many of the subjects (72%) suffered from a medium level of BO, 7% suffered from a high level of BO, and only 21% had low levels of BO.

We found it worthwhile to compare the levels of depressive and anxiety symptoms and BO according to the work/intervention modes since October 2020, i.e., during the period of partial lockdown. During this period, most professionals worked face-to-face and in alternating shifts, and few worked exclusively at a distance (see above). As mentioned in Table 4, our results showed that there was no significant difference in depression and anxiety between these different modes. On the other hand, professionals working face-to-face had the highest scores on the “exhaustion” subscale of the BO. There was no significant difference between disengagement and total BO. Concerning the participants’ patients, the comparative analyses according to the level of precariousness of the patients showed significant differences between groups, with those working mainly with patients in precarious situations presenting significantly higher scores of BO and exhaustion.

#### 3.3.1. Problematic Situations and Difficulties in the Professional Environment

In the survey, psychologists answered the next question: ‘Name 1 to 3 problem situations or difficulties experienced in your work since the COVID-19 pandemic?’

Five major themes emerged from the analysis of the first problematic situations (see Table 5) based on 163 answers obtained:

(1) Barrier gestures and safety distance were experienced as obstacles to communication and clinical work: The entrance of health measures was described by psychologists as an obstacle to relational work. The obligatory physical distance created a relational distance with patients, and the mask made expression and observations of emotions difficult.
(2) Distance and telework cause issues at different levels, according to the psychologists:
  - Organizational level: Communication problems, paradox, and institutional organization.
  - Clinical level: Dealing with emotional consequences of the crisis (e.g., social isolation, suicidal attempts) and keeping the social link even remotely by adapting.
  - Team level: Maintain a team dynamic remotely and the lack of contact with colleagues.
  - Network level: Loss of contacts with the network’s partners.
(3) Radical change in the workload: Some psychologists described an increase in requests, and others described a full stop or a reduction of their activities in a few days.
(4) Reach and help people at a distance: Face to the first lockdown, a lot of professionals felt helpless in the face of patients’ suffering, not knowing how to intervene with them remotely, and being confronted with computers, technical problems, and a lack of resources.

#### 3.3.2. Motivating and/or Positive Situations in the Professional Environment

As with the previous question, psychologists gave 161 answers to the first motivating situation at work during the COVID-19 pandemic (‘Name 1 to 3 motivating and/or positive situations you have experienced in your work since the COVID-19 pandemic?’). Seven main themes emerged from the first motivating situation’s analysis (see Table 6):

1. Strengthening solidarity and mutual support between colleagues during the crisis: Psychologists described an increase in mutual support between colleagues and a strengthening of team cohesion in the face of difficulties. Some of them also reported listening, support, and encouragement from their direction.
2. Reinforcement of helping relations and intensification of collaborations to be present for patients: Great importance is given to maintaining the services open despite the lockdown to be present for patients thanks to the intensification of collaborations and more individual’s time.
3. Increasing of usefulness or responsibility feelings and frontline role: The COVID-19 crisis strengthened the sense of usefulness, societal responsibility, and the frontline role of psychologists during the COVID-19 pandemic. These feelings were notably reinforced through positive feedback and recognition from the patients about their presence and help during the lockdown.
4. Sharing of experiences and collaborations between professionals in the COVID-19 context: The sharing of practices motivated professionals to increase collaborations, exchanges, and meetings between them to deal with the COVID-19 context.
5. Adaptation, creativity, and new practices in health crisis context. In this unprecedented situation, psychologists developed adaptive capacities and creativity to provide care to patients, build new projects, and reinvent their practices with new means of communication (e.g., phone and videoconference).
6. Relaxation of the employer’s framework, flexibility, and more openness toward creative projects: The obligation to telework has brought a reduced workload, less stress, and better management of private and professional time.
7. None: This theme means that they had not experienced any motivating situations during the COVID-19 pandemic.

#### 3.3.3. Practices Developed during the Pandemic

In the questionnaire, an item was dedicated to new practices developed by psychologists during the COVID-19 pandemic (i.e., Could you describe 1 to 3 practices that you have developed since the COVID-19 pandemic?’). The first practice described has been analyzed based on 164 answers. The interventions described by the respondents can be grouped into 4 categories (see Table 7):

(1) Remote interventions and use of digital media: Many psychologists mentioned using internet resources and digital media for different purposes, mainly with the aim of maintaining and optimizing communication within services, networks, and for patients. They also report the transition to individuals, families, and groups in teleconsultations.
(2) Proactive interventions “outreaching” and flexibility: Many psychologists described a change in their roles as psychologists and in their framework of interventions. They began to take proactive approaches, where they made and maintained contact with patients and visited them in their living places. All these types of interventions involved therapists reaching out to patients and anticipating their needs. This proactive approach is achieved through a diversification of intervention modalities and an expansion of the usual framework (more flexibility, increased frequency of contact, and home consultations).
(3) Innovative and creative interventions: The third category includes the development of interventions and therapeutic media that reflect the creativity of the psychologists surveyed. It concerns their strategies, developed constraints, and impacts of the crisis to ensure consultations with patients who were not accessible by video or who were withdrawn. In addition, this theme includes the creation of tools as support to manage stress and emotional consequences linked with lockdown, as well as coming up with solutions such as walking therapies.
(4) Social interventions for disadvantaged people: Participants indicated that they had integrated more social missions into their work by providing support in the establishment of social assistance programs and by organizing and transporting essential equipment and goods. The pandemic has clearly had an impact on the usual framework of interventions, and all the practices reported in the survey reflect the concerns of these professionals to maintain patient follow-up and their willingness to overcome obstacles generated by health measures to meet the perceived needs of the population.

## 4. Discussion

The COVID-19 pandemic and the health measures put in place to contain it have strongly affected the well-being of the general population, causing psychological distress, the levels of which increased throughout the crisis. As a result, mental health needs have intensified and diversified. At the same time, psychological care services have had to adapt to the measures prescribed at different stages of the crisis, which may have had an impact on the mental health of MHP, as observed among other caregivers [13]. However, mental well-being is a key determinant of MHP’ ability to provide high-quality care [46,47] and should be considered in policies developed during the pandemic. To this end, our study focused on professionals working in the field of psychology, and our analysis is based on a sample of 187 psychologists in French-speaking Belgium working in a variety of institutional contexts and with a variety of patients. Our results provide a nuanced picture. The majority of respondents perceived a negative effect on their mental health, and half of them declared themselves discouraged and demotivated. More than 70% have suffered from professional burnout. However, our results also suggest that most of the psychologists surveyed did not experience high levels of depression or anxiety. Indeed, the prevalence rates (17%; 12%) and average scores on the depression and anxiety scales are lower than those obtained in a study in the general population (18%; 20%) and among students in higher education (55%; 51%) in Belgium [9,10]. These results are consistent with Brillon’s [14] study, which also found lower rates of depression and anxiety among MHP when compared to other workers in the general population. The prevalence of psychologists also appears to be lower than those found in the meta-analyses of healthcare workers during the COVID-19 pandemic (40% (95% CI: 29–52%), 37% (95% CI: 29–45%) [11,14,48]. Our results concur with those of Minelli et al. [48], who found that psychologists/psychotherapists and psychiatrists were less affected than other mental health professions. This suggests that psychologists, by virtue of their profession, are more likely than other professions, even those in the medical sector, to mobilize resilience and adaptation strategies that are better suited to coping with stress, constraints, and demands during a crisis. They are also more likely to use resources, such as supervision and psychotherapy, which was noted prior to the crisis context [49,50,51]. Moreover, half of the psychologists in our study reported stronger feelings of professional commitment, taking to heart the demands—which increased for a third of the sample—as well as the perceived needs of more vulnerable populations, including both children and adults. They also developed new frameworks and modes of intervention in order to ensure patient follow-up. In the context of lockdown in particular, psychologists made proactive interventions, such as phoning the patient on a weekly basis, visiting the living environment, and mobilizing resources and the network around the patient, among others. The fear of losing links with patients in the context of crises has motivated psychologists to adopt more proactive approaches in their work. In addition, psychologists felt more useful and effective in their interventions, and felt more effective in being proactive in responding to patient distress with the usual methods of intervention to respond to patient distress.

To be considered proactive, these types of approaches must be undertaken before or without a formal request for services from the individual [52], and they refer to a model of intervention characterized by a decentralization of services to communities and a greater proximity between MHP and the citizens. Given the known barriers to recourse to psychological assistance, this preventive approach should also facilitate access to care and early intervention [53].

In the context of a pandemic and the confinement of individuals, mobility, and the expansion of the therapeutic approach and framework are means of ensuring that individuals receive support. Therapeutic distance in the context of social distancing was renegotiated to ensure continuity of care through proximity and to offer supportive and sufficiently reassuring care. In this way, a widening of the usual framework of intervention was established and legitimized by institutions offering a care experience that was perceived by the MHP as innovative and motivating. Although these data show resilience built on a commitment to providing care, psychologists were not spared from professional burnout; 7 out of 10 psychologists in our study suffered from medium BO, and 7% from high BO. These prevalences are even higher than those found in recent systematic reviews of healthcare workers [54]. These studies showed that almost half of healthcare workers suffered from burnout during the COVID-19 pandemic. The overall pooled prevalence of burnout was 52% [95% confidence interval (CI) 40–63%]. The combined prevalence of emotional exhaustion (EE), depersonalization (DP), and lack of personal accomplishment (PA) was 51% (95% CI 42–61%), 52% (95% CI 39–65%) and 28% (95% CI 25–31%), respectively [54]. However, the other systematic review on the prevalence and causes of BO conducted prior to the COVID-19 pandemic already noted that burnout is a concern for those working in the delivery of psychological interventions. Emotional exhaustion is the most frequently reported dimension of burnout, with professional and personal characteristics and resources also playing an important role in the development of burnout in the mental health care profession [55]. The high rates of exhaustion in our study (75% medium and 11% high exhaustion) are consistent with the data from this systematic review and suggest that BO for psychologists, as for other health workers, has been exacerbated by the impacts of the crisis. Longitudinal studies to follow the evolution during and after the crisis of mental health and the burn-out of mental health workers and psychologists should be supported and conducted in Belgium and other countries.

The interest of our study was to analyze the different variables of psychologists’ mental health according to the modes of work (remote, hybrid, and face-to-face) and the patients’ social vulnerability profile. After a year of the COVID-19 pandemic, which included periods of complete and partial lockdown, professionals transitioned, according to the measures, between different modes of remote, face-to-face, and hybrid clinical services. Unsurprisingly, it was during the period of strict lockdown (March–May 2020) that the most remote interventions were reported (50%; 13% alternated between telework and on-site work, and more than a quarter (28%) continued their services on site). As soon as the measures were lifted, the majority (70%) resumed their face-to-face services, and a quarter alternated between telework and remote consultations, with an increasingly pronounced downturn in telework and remote consultations during the subsequent stages of the pandemic. Thus, very few (2%) of the psychologists in our study continued to work remotely (by video or telephone) after a year of crisis, 65% resumed their face-to-face monitoring and consultations with barrier gestures, and 33% continued to alternate between face-to-face and remote consultations. These transitions show a return-to-field practices favoring face-to-face interventions, even though these are a risk factor for professional exhaustion. In fact, our results show that psychologists who have returned to face-to-face clinical care “in the field” in the institution or in the living environment are more likely to suffer from burnout. This can be explained by the stress generated due to direct confrontation with patients’ distress, the complexity of the situations faced, the difficulties encountered in offering the necessary support in view of the number of requests received, and issues related to networking with professionals. Moreover, it is possible that video consultations, based on the patient’s narrative, do not allow for the same perception of needs as face-to-face consultations and could even hide signals and indicators of the difficulties they face. While the pandemic has dramatically accelerated the adoption of telepsychology and the adherence to these practices by psychologists—which should indeed be among the “tools” in different contexts [56]—the limitations of these practices should also be noted. Among these are the risks of not meeting the needs of different populations and of not allowing professionals adequate access to the complexity of situations, particularly for the most precarious populations, as they may not actually have access to the equipment necessary for telepsychology and, therefore, end up being deprived of this form of support. Moreover, these populations need to be in contact with professionals (meetings, links, and third parties) to make their requests [57], which requires a proactive approach, as the professionals in the study have testified. However, this is not without difficulty. Indeed, our results show that psychologists working with disadvantaged populations are the ones who are most affected by professional burnout. The unstable living conditions and numerous difficulties that these patients usually face have been exacerbated by the crisis and have led to extreme situations. These families are at greater risk of being exposed to COVID-19 infection because of their living conditions, and of losing their often-precarious jobs without being able to satisfy their basic needs [58]. Faced with this psychosocial distress, professionals have developed new proactive intervention strategies and have invested in social aid missions to meet the most basic needs of their patients while running the risk of becoming emotionally exhausted, which they express with discouragement, feelings of powerlessness in the face of these situations, and a lack of resources. Policies of material support, personnel means (in terms), and quality supervision must be reinforced for psychologists.

## 5. Limitations

This research has certain limitations. First, the sample is based on an online survey and is not representative of all psychologists; in particular, self-employed psychologists were under-represented in our sample. However, our sample included psychologists working in different sectors and services (Table 3). The research focused on the profile of patients, mode of intervention, and perceived socio-economic status of patients during the crisis but did not take into account variables that have been identified in previous research as having an impact on mental health in times of crisis, such as job insecurity, economic uncertainty, and professional uncertainty. Future studies could incorporate these variables to measure the impact on the mental health of psychologists two years after the onset of the pandemic crisis [59,60,61]. Another variable that was not included in the quantitative study was the support that institutions provided to psychologists (facilities, recognition, and relationships with the hierarchy). Indeed, recent research has shown that organizational support is a protective factor in relation to professional stress [62], as is good-quality supervision [63,64,65], which can reduce burnout among MHP. Future research on psychologists in times of pandemic crisis should be supported, as data on these professionals are scarce in Belgium, as in other European countries. In addition, longitudinal data at different periods of the COVID-19 crisis (after two years) and at the end of the pandemic would report on the evolution of mental health and the possible sustainability of psychological practices developed during the COVID-19 pandemic. It would be interesting for future research to focus on organizational variables and other supports that support the well-being of psychologists.

## 6. Conclusions

The COVID-19 pandemic and the health measures adopted in response to it have affected the care framework and practices of psychologists, without having too great an impact on their mental health, as has been observed in other medical professions. Psychologists have most certainly mobilized skills and coping strategies developed in the context of their profession. The fear of weakening relationships with patients (but also with colleagues and partners in the network), as well as their commitment to providing care [66], are at the heart of these professionals’ concerns. In the context of the current pandemic, they have benefited from greater flexibility. Due to the urgency with which they had to respond to the needs of the population, the management of the institutions validated new and different types of interventions carried out by psychologists. They have broadened their framework of interventions through proactivity and have developed new therapeutic supports and media (such as walking therapy, stress management workshops, and relaxation exercises), which have been a source of motivation and positive stimuli for them. Digital media have been useful both for teleconsultations and the establishment of digital communication spaces within teams and the wider professional network, and this has resulted in new experiences and the development of new skills that are likely to be re-inscribed in current practices, provided that the proper uses and misuses are considered. It is clear that psychologists have shown resilience despite the lack of recognition from public authorities. The recognition of their efforts by patients and their hierarchy has partly compensated for the official silence surrounding their actions. However, our research has shown that one year after the beginning of the crisis, these professionals are experiencing professional exhaustion, especially in the context of interventions with the most vulnerable and socially precarious populations, the size of which is likely to increase throughout the pandemic and post-pandemic periods. The COVID-19 crisis exacerbated social inequalities, and the most vulnerable before the crisis were the most affected by it. Support, inclusive and holistic assistance programs, and measures should be strengthened to reduce the psychological distress of this population and the MHP working in this sector.

## Figures and Tables

**Table 1:** Cut-off scores to interpret results for the Oldenburg Burnout Inventory (OLBI) and Hospital Anxiety Depression (HAD).

Scales	Subscales (Items)	Cutoff Scores		
		**Low**	**Medium**	**High**
**OLBI**	*Total*	<30	30–44	>44
	*Exhaustion (8)*	<16	16–23	>23
	*Disengagement (8)*	<15	15–22	<30
**HAD**		**No symptoms**	**Suspected symptoms**	**Proven symptoms**
	*Depression (7)*	≥7	8–11	>11
	*Anxiety (7)*	≥7	8–11	>11

**Table 2:** Prevalence rate for social professionals’ characteristics of the psychologists’ sample (N = 187) and their working environments.

	Before the COVID-19 Pandemic	First Lockdown March–May 2020	Between Both Lockdowns June–September 2020	Second Lockdown October–April 2021
*On site*	75%	27%	70%	55%
*Teleworking*	3%	50%	4%	35%
*Both*	22%	13%	25%	9%
*Off work*		10%	1%	1%

**Table 3:** Prevalence rate for social professionals’ characteristics of the psychologists’ sample (N = 187) and their working environments.

Working Environments	% (N = 187)
Type of services	
*Youth protection*	26%
*Mental Health Centers*	23%
*Psychosocial services*	22%
*Hospital*	18%
*Penal institutions*	6%
*Mobile team*	5%
Patient’s population	
*Adults*	63%
*Teenagers*	16%
*Children*	21%
Perceived socio-economic status	
*Various social and economic profiles*	49%
*Mostly socially precarious*	40%
*Highly precarious socially and economically*	11%

**Table 4:** Prevalence—Average HAD and OLBI scores and intergroup comparisons by work arrangements and socio-economic status.

HAD Scale N = 187	No Symptoms % (n)	Suspected Symptoms % (n)	Proven Symptoms % (n)	Mean (SD) Total Sample	Mode of Interventions. * Mean (SD) Comparison	Perceived Socio-Economic Status of Patients ** Mean (SD) Comparison
**Depression**	**61.5** (115)	**21.9** (41)	**16.6** (31)	6.71 (3.67)	6.75 (4.78) 6.55 (3.83) 7.02 (3.32) **ns**	6.33 (3.10) 6.78 (4.05) 8.20 (4.31) **ns**
**Anxiety**	**63.6** (119)	**24.1** (45)	**12.3** (23)	6.65 (3.24)	4.25 (1.89) 6.81 (3.43) 6.50 (2.86) **ns**	6.60 (3.11) 6.64 (3.51) 6.90 (2.88) **ns**
**OLBI Scale** **N = 187**	**Weak**	**Medium**	**High**	**Mean (SD)** **Total Sample**	Mode of Interventions. * **Mean (SD)** **Comparison**	Perceived Socio-Economic Status of Patients ** **Mean (SD)** **Comparison**
**Exhaustion**	**14.4** (27)	**74.9** (140)	**10.7** (20)	18.53 (4.09)	13.25 (4.92) 18.80 (4.12) 18.33 (3.78) **F = 0.024** **2 > 3 > 1**	17.78 (4.24) 18.97 (3.71) 20.30 (4.15) **F = 0.021** **3 > 2 > 1**
**Disengagement**	**24.1** (45)	**69.5** (130)	**6.4** (12)	16.60 (4.24)	16.25 (3.06) 16.68 (4.24) 16.48 (4.36) **ns**	16.05 (4.14) 16.86 (4.33) 18.15 (4.06) **ns**
**BO Total**	**21.4** (40)	**71.6** (134)	**7** (13)	35.14 (7.53)	29.50 (7.72) 35.49 (7.63) 34.82 (7.53) **ns**	33.84 (7.61) 35.83 (7.17) 38.45 (7.48) **F = 0.026** **3 > 2 > 1**

* Mode of work: 1 = distance; 2 = face-to-face; 3 = alternating. ** Perceived socio-economic status of patients: 1 = diverse socio-economic background; 2 = precarious/vulnerable; 3 = very precarious.

**Table 5:** Thematic analysis of the first problematic situation described by psychologists in the professional environment for the COVID-19 pandemic.

Main Themes (n = 162)	Sub-Themes	Verbatim Examples
**1. Barrier gestures and safety distance experienced as obstacles to communication and clinical work**	Barrier gestures and the safety distance create a relational distance	*“Contact behind plexiglass with mask, creating distance from people.”* *“Difficulty with barrier gestures with emotionally demanding children.”*
	Wearing a mask hides the face, impairs non-verbal communication and emotional expression	*“Working with the mask hides the emotions of the face.”* *“Wearing the mask handicaps communication.”*
	Undermining of relational work due to physical distance	*“Not able to help people as well because of difficulties with face-to-face.”* *“Working on interactions without being in interaction...”* *“Lack of physical and/or visual contact which is helpful in creating a bond more easily.”*
**2. Distance and telework causing issues at different levels:**	**Organisational level:** Communication problems, paradox, and institutional organisation	*“Organizational difficulties following hybridization.”* *“Permanent changes in the organisation of the service.”* *“Paradoxical demands from the hierarchy.”*
	**Clinical level:** Dealing with emotional consequences of the crisis	*“Increase in suicidal acts and attempts.”* *“Social isolation of patients.”* *“Increased severity of clinical situations.”*
	**Clinical level:** Keep the social link even remotely by adapting	*“Maintaining the link with users.”* *“Having to rethink our work at a distance (first lockdown).”* *“Changing the framework of intervention by becoming very proactive.”*
	**Team level:** Maintain a team dynamic remotely	*“Great difficulties in clinical exchanges during video meetings.”* *“Uncertainty to be managed to contain the teams.”* *“Lack of presence of colleagues for team meetings—quality of exchange is lost!”*
	**Team level:** Lack of contacts with colleagues	*“It is difficult to pass on information in an informal setting. Everyone stays in their own office, especially during breaks.”* *“Lack of contact with colleagues and therefore of informal exchanges so precious!”* *“Loss of contact with colleagues / less fluid communication / loss of team spirit.”*
	**Network level:** Loss of contacts with network’s partners	*“Fewer stakeholder meetings around a situation.”* *“Loss of connection with professionals.”* *“Efficiency decreased or inaccessible care network due to rules.”*
**3. Radical change in the workload**	Increasing of requests	*“The ever-increasing number of cases.”* *“A lot of requests.”* *“Considerable increase in the workload.”*
	Stop or reduction of activities	*“Strong decrease in the number of patients.”* *“No longer able to welcome our public.”* *“The lack of people presents during the activities.”*
**4. Reach and help people at a distance**	Powerlessness feeling	*“Feeling of powerlessness during the first lockdown because of the impossibility of physically seeing the beneficiaries.”* *“Loss of contact with the public. The more time passes, the less young people respond via networks, messages, calls... What else can I do?”* *“Hearing the suffering of people excluded from society without being able to act.”*
	Technical, IT and lack of resources	*“Digital divide: some people do not have access to the necessary equipment for video interviews.”* *“Lack of space, sometimes a headache to arrange an interview.”*
**5. Weakening of the boundary between private and professional life**	Distinction between work and private life in teleworking	*“Blurring the boundary between private and professional life with teleworking.”* *“The distinction between work and private life when I telework.”*
	Management of children and work	*“Difficulties in working at home when primary school children were no longer in school.”* *“Teleworking with 2 children to educate oneself.”*

**Table 6:** Thematic analysis of the first motivating situation described by psychologists in the professional environment for the COVID-19 pandemic.

Main Themes (n = 161)	Sub-Themes	Verbatim Examples
**1.** **Strengthening of solidarity and mutual support between colleagues during the crisis**	Increased mutual support and solidarity between colleagues in the face of difficulties	*“Strengthened links with colleague’s support to cope together with the current extreme psycho-social situation.”* *“Increased solidarity between colleagues.”* *“More email exchanges with colleagues, some obvious support.”*
	Strengthening of team cohesion with the health crisis	*“Strengthened team links.”* *“Solidarity within teams facing a crisis situation.”* *“The observation of good team cohesion, a supportive team.”*
	Listening, supporting, and encouraging its management in the face of the health crisis	*“Management support and encouragement.”* *“Listening carefully to the management.”* *“The encouragement of the hierarchy, where we regularly received emails in this sense and thanking us.”*
**2.** **Reinforcement of helping relation and intensification of collaborations to be present for patients**	Keep the services open despite the lockdown to be present for patients	*“Finding solutions in very critical situations.”* *“Ensure continuity of care against all odds.”* *“Even remotely, sessions are important for patients.”*
	Intensification of collaborations and more individual times	*“Close collaboration with some general practitioners closes to young people and their families.”* *“Very positive collaborations within schools to support young people and parents.”* *“More time to spend individually.”* *“Individual activities.”*
**3.** **Increasing of usefulness or responsibility feelings and frontline role**	Strengthening the sense of usefulness or responsibility and the frontline role	*“Sense of usefulness despite frustrations.”* *“Reinforcing the importance of our frontline role.”* *“Reassessment of the need for psychologists to deal with psychological distress.”*
	Positive feedbacks and recognition from patients for presence and help during the crisis	*“Positive feedback from my patients about the help I give them.”* *“Patients’ recognition of professionals.”* *“Relief to patients that we are always open.”*
**4.** **Sharing of experiences and collaborations between professionals**	Increased collaboration, exchanges, and meetings to share practices between colleagues/partners	*“Positive mobilization of resources between colleagues.”* *“Collaboration with GP and psychiatrist more accessible.”* *“Greater mobilization of the network for situations requiring it.”*
**5.** **Adaptation, creativity, and new practices in health crisis context**	Activating the adaptive capacities of professionals and creativity to provide care to patients	*“Coping with novelty.”* *“Adaptations and innovations that stimulate.”* *“The constant need for creativity.”* *“A lot of space given to creativity in order to continue the support.*
	Building new projects in the context of the health crisis	*“Creation of new projects for the post-covid era.”* *“Creation of a Zen workshop.”* *“Specific support projects for the most vulnerable in the context of the COVID-19 crisis.”*
	Reinventing practices, formations and seeking new means of communication	*“Adaptation of the team to the use of new technologies (video consultation, WhatsApp).”* *“Learning and creating new ways of doing things.”* *“The possibility of taking time out to reflect on one’s practice and read.”*
**6.** **Relaxation of the employer’s framework, flexibility, and more openness towards creative projects**	Reduced workload, stress, and more flexibility	*“Flexibility of my employer.”* *“I had less work to do, less stress to deal with, less mental and time pressure on my days.”*
	Better management of private and professional time when teleworking	*“Telework facilitates personal time use.”* *“More flexibility in my work and family schedules.”*
**7.** **None**		*“None.”* *“I don’t see any.”* *“I have no answer.”*

**Table 7:** Thematic analysis of the practices developed by psychologists during the COVID-19 pandemic.

Main Themes (n = 164)	Sub-Themes	Verbatim Examples
**1.** **Remote interventions and use of digital technology**	Telephone calls, WhatsApp, Messenger, Skype, SMS	*“WhatsApp with users.”* *“Messenger interview with young people.”* *“Telephone links with housebound youth.”*
	Individual, family, and group consultations by video	*“Visits to children and dads by video.”* *“Use of video conferencing for family interviews.”* *“Focus groups by video.”* *“Session with patient by phone.”*
	More frequent use of emails to communicate with patients and network	*“Communicate much more by e-mail, telephone with beneficiaries and the work network.”*
	Creation of digital platforms and internet sites	*“Creation of a forum and Facebook page”; “Communication and support to young people and families through multimedia communication tools.”*
**2.** **Proactive interventions “outreaching” and flexibility**	Regular contact with patients (e.g., weekly calls, old patients)	*“More regular telephone contact.”* *“Re-establishing contact with former monitors who are experiencing temporary difficulties.”*
	Intensification of frequency and regularity of meetings with patients	*“During the first lockdown (March-May 2020), we set up weekly telephone appointments, and more, if necessary, with the families for whom we were commissioned.”* *“Reorganization of the frequency of sessions and the clinical agenda.”*
	Support and feedbacks reinforced by teams	*“Stress management support workshop.”* *“24-hour telephone hotline to support colleagues.”* *“Increased support and listening to educational teams.”*
	Authorization of last-minute cancellations without charge	*“Allow last minute cancellations without charge.”*
	Reaching out to patients	*“Door-to-door activities at beneficiaries’ homes.”* *“Meeting children in schools.”* *“Outdoor home visit only.”*
**3.** **Innovative and creative interventions: development of new interventions and therapeutic tools**	Walking therapy	*“Walking therapies”* *“Walking with patients”* *“Therapeutic walk.”*
	Use of writing as a therapeutic tool	*“Use of writing with prisoners.”* *“Epistolary psychotherapy: mail exchanges with my patients.”*
	Creation of tools/materials to aid the expression of emotions during remote sessions	*“A Zen workshop for young people (sophro, relaxation...).”* *“Stress management support workshop.”* *“Suggested relaxation exercise in interview.”*
	Focus groups considering experiences during the pandemic	*“Creation and proposal of an animation about the coronavirus (adapted to the age of the class groups): info + management.”* *“Focus groups on COVID or vaccination.”*
	Development of specific psycho-education modules related to the pandemic	*“Development of specific psycho-education modules related to the pandemic.”*
**4.** **Social interventions to disadvantaged people**	Social support for the most precarious populations	*“Daily phone calls to isolated people.”*
	Participation in the distribution of necessities (e.g., food assistance, hygiene materials, etc.)	*“Distribution of meals at home.”* *“Distribution of meal vouchers and basic materials (toilet paper, paper towels, soap, feminine hygiene products, washing-up liquid, laundry products...).”*
	Social services and support	*“Establishment of an official collaboration with the non-profit organization providing food box.”* *“Opening of a support line accessible and free to all.”*

## Data Availability

The data presented in this study are available on request from the corresponding author. The data are not publicly available due to ethical restriction.
